# Factors Associated with Dropout Intention in Engineering Education: A Learning Interpretable Modeling Approach

**DOI:** 10.3390/bs16050717

**Published:** 2026-05-07

**Authors:** Liliana Pedraja-Rejas, Nayeli Ocaranza, Pamela Ocaranza Paz, Laura Perez

**Affiliations:** Departamento de Ingeniería Industrial y de Sistemas, Facultad de Ingeniería, Universidad de Tarapacá, Arica 1000000, Chile; nayeli.ocaranza.araya@alumnos.uta.cl (N.O.); pamela.ocaranza.paz@alumnos.uta.cl (P.O.P.); lperez@academicos.uta.cl (L.P.)

**Keywords:** dropout intention, student retention, engineering education, logistic regression, decision tree, higher education

## Abstract

Dropout in higher education remains a persistent challenge with academic, institutional, and social consequences. Identifying students’ dropout intention before formal withdrawal occurs may provide useful evidence for preventive support strategies. This study examines dropout intention among undergraduate engineering students at a Chilean university. Using survey data from 189 students, five variables were operationalized: personal self-regulation, mental health, institutional perception, socio-economic conditions, and academic motivation. Two interpretable models were estimated: multivariable logistic regression and a shallow decision tree. The logistic regression model showed satisfactory explanatory capacity and identified socio-economic conditions and personal self-regulation as significant protective predictors of dropout intention, while mental health, institutional perception, and academic motivation were not significant after adjustment. The decision tree provided a complementary rule-based segmentation and confirmed the prominence of socio-economic conditions and personal self-regulation, while also indicating a secondary contribution of mental health. These findings suggest that dropout intention in engineering education is shaped primarily by structural conditions and self-regulatory resources rather than by institutional perception or academic motivation alone. The study provides empirically grounded evidence to support early identification and student support strategies in higher education.

## 1. Introduction

Student dropout in higher education is a persistent challenge that affects educational quality, academic performance, and institutional sustainability. Beyond its immediate academic consequences, dropout generates financial losses for institutions, weakens human capital formation, and deepens social inequalities associated with unequal educational trajectories. Because of its multidimensional impacts, understanding not only the occurrence of dropout but also its antecedent processes has become a central concern for higher education research and policy.

Within this broader understanding, increasing attention has been paid to students’ dropout intention as an antecedent indicator of effective withdrawal. Unlike administrative records that capture dropout only once it has occurred, intention-based measures make it possible to detect early signals of disengagement before withdrawal becomes irreversible. This anticipatory perspective enables earlier identification of at-risk students and opens a window for preventive institutional action. Research on student retention and attrition shows that the risk of withdrawal is shaped not only by academic performance but also by psychosocial conditions, mental health, socio-economic constraints, and students’ experiences and integration within institutional environments (Matz et al., 2023; Monaghan & Sommers, 2022; Shafiq et al., 2022; Valencia-Arias et al., 2026; Villegas-Ch et al., 2023). Extending this perspective to the attitudinal domain, dropout intention can be understood as a proximal manifestation of these same structural and experiential pressures, reinforcing the need for integrative analytical approaches capable of capturing their relative and combined effects.

Despite the expansion of predictive approaches to student retention, much of the empirical evidence remains concentrated in performance-centered explanations or in single-domain models that underestimate the joint influence of psychosocial and institutional determinants. Furthermore, while predictive accuracy has improved substantially, interpretability has not always received equivalent attention, limiting the capacity of universities to translate statistical outputs into actionable student support strategies. Understanding not only whether a domain matters but how strongly it matters and under what conditions is essential for designing targeted retention policies grounded in empirical evidence. At the same time, this is also an institutional challenge, since the value of such evidence depends on how universities interpret it and translate it into organizational responses.

From an organizational learning perspective, this challenge extends beyond the statistical identification of student risk. Building on the institutional learning model proposed by Rodríguez-Ponce et al. (2024), academic outcomes may be understood as being shaped by the university’s capacity to acquire knowledge, distribute and interpret information, and develop organizational memory. Within this broader framework, dropout intention may be interpreted as a teaching-related outcome expression of the university’s capacity to sustain students’ academic trajectories. In turn, as Labraña and Rodríguez Ponce (2026) suggest, the institutional value of predictive and data-driven tools depends not only on their technical performance but also on how universities process the information they generate through organizational learning and decision premises. Interpretable evidence on dropout intention is therefore relevant not only for early identification but also for strengthening institutional learning about student support and retention practices.

Addressing these gaps, this study adopts an integrative perspective to examine dropout intention among undergraduate engineering students at a Chilean university. Drawing on a survey of 189 students, five variables were operationalized: personal self-regulation, mental health, institutional perception, socio-economic conditions, and academic motivation. In this context, the study seeks to answer three interrelated questions:

**RQ1:**  *Which variables are significantly associated with students’ dropout intention?*

**RQ2:**  *Which variables show the strongest adjusted association with dropout intention?*

**RQ3:**  *How can the lack of statistical significance of some theoretically relevant variables be plausibly interpreted in the estimated multivariable models?*

By addressing these questions, the article contributes empirical evidence based on interpretable models regarding the determinants of dropout intention in engineering education. More specifically, it provides comparative insight into the relative explanatory weight of psychosocial, socio-economic, institutional, and motivational variables.

## 2. Background

Dropout intention has been conceptualized as a proximal indicator within broader models of student persistence and withdrawal. Unlike observed dropout, which represents the final stage of disengagement, dropout intention refers to an attitudinal and decisional state that may precede formal withdrawal and therefore offers an analytically useful window into the mechanisms underlying persistence. Within higher education research, this perspective has been shaped by established theories of student departure and retention. Spady (1970) first framed dropout as a process related to students’ integration into the normative and social life of the institution, while Tinto (1975) developed a more systematic explanation linking persistence to academic and social integration. J. P. Bean (1980) placed greater emphasis on attitudinal processes and intention to leave, and J. P. Bean and Metzner (1985) further showed that external conditions such as finances, work demands, and environmental pressures may weigh especially heavily in students’ withdrawal decisions. More recent integrative work argues that these perspectives are best understood together, since students’ persistence is shaped by the interaction of institutional experience, individual adjustment, and external constraints (Cabrera et al., 1993; Hadjar et al., 2023).

A growing body of research shows that psychosocial strain and well-being are closely associated with students’ persistence trajectories. Perceived overload, chronic stress, and reduced coping capacity can undermine engagement and increase withdrawal considerations (e.g., Crockett et al., 2024; Véliz Palomino et al., 2024). These findings align with broader evidence indicating that psychological distress and mental health difficulties can weaken academic integration and persistence. In this sense, mental health is relevant not only as an individual condition but also as a dimension that shapes how students experience academic demands and sustain their commitment to their studies. This broader view resonates with psychological approaches to retention that emphasize coping, adjustment, and students’ capacity to manage stress as part of the processes supporting persistence (J. Bean & Eaton, 2001).

Socio-economic conditions constitute another central domain in persistence research. Financial constraints, the need to combine work and study, and limited access to material resources can increase time pressure and reduce academic availability, thereby heightening vulnerability to dropout (Aina et al., 2022; Lamichhane et al., 2025). Empirical studies consistently show that financial stress and employment intensity are associated with lower retention rates and greater discontinuation risk. These structural pressures may also interact with psychosocial strain, reinforcing cumulative disadvantage processes that contribute to dropout intention. This domain is therefore especially relevant in approaches that emphasize the role of external environments and competing obligations in shaping students’ continuation decisions (J. P. Bean, 1980; J. P. Bean & Metzner, 1985).

At the individual level, personal self-regulation has been identified as a protective factor in higher education trajectories. Goal clarity, planning capacity, persistence, and adaptive coping can contribute to sustained academic engagement (Bardach et al., 2020; González & Arce, 2021). Self-regulation may help students manage competing demands, organize study time, and maintain effort under pressure, thereby reducing vulnerability to disengagement. From a psychological retention perspective, these capacities can be understood as part of the internal resources through which students respond to institutional and academic demands (J. Bean & Eaton, 2001). At the same time, in multivariate settings, self-regulation may overlap conceptually with related domains such as motivation, making empirical comparison necessary.

Institutional perception also matters for persistence decisions. Students’ evaluations of teaching quality, curriculum coherence, administrative processes, and institutional support structures can influence their sense of belonging and academic commitment (Marca Maquera et al., 2025; Sanhueza Gutiérrez et al., 2021). However, the magnitude of these effects may vary across contexts and may be partly conditioned by personal and psychosocial factors. Rather than treating institutional perception as an isolated determinant, it is more appropriate to examine it together with other domains that shape students’ experience of the educational environment and their academic continuity (Hadjar et al., 2023; Tinto, 1975).

Academic motivation and engagement are likewise often described as central drivers of persistence. Effort investment, goal orientation, and professional projection have been linked to continued enrollment (Lorenzo-Quiles et al., 2023). Research in STEM education also suggests that learning environments may contribute to the development of career aspirations and professional projection, thereby reinforcing students’ future-oriented engagement (Mahmood et al., 2025). Even so, motivation should not be assumed to operate independently of other psychological, institutional, and structural conditions. Its contribution is better evaluated empirically alongside related domains, particularly in multivariate models where some variables may show stronger net associations than others (J. P. Bean, 1980; J. Bean & Eaton, 2001).

Taken together, the literature suggests that dropout intention reflects the interplay of structural, institutional, psychosocial, and individual influences. Although these domains have been addressed in prior research, they are less often examined simultaneously through interpretable multivariable models. In this context, the present study analyzes five variables—personal self-regulation, mental health, institutional perception, socio-economic conditions, and academic motivation—in relation to dropout intention among undergraduate engineering students.

## 3. Materials and Methods

A non-experimental, cross-sectional research design with a quantitative approach and descriptive–explanatory scope was adopted, consistent with established social science research design frameworks (Creswell & Creswell, 2023). This design is appropriate for examining relationships between variables as they naturally occur, without manipulation or experimental intervention. The study relies on survey-based measurements to identify patterns, associations, and predictive relationships between psychosocial, institutional, and socio-economic factors and students’ dropout intention. In addition to its descriptive and explanatory components, the study incorporates a predictive analytical dimension through the application of binomial logistic regression and decision tree modeling.

### 3.1. Participants and Data Collection

Participants were undergraduate students enrolled in the face-to-face Industrial Engineering program at a university located in northern Chile. The study population comprised 625 students registered in 2025. Sample size was calculated using the finite population formula, assuming a 90% confidence level, a 5% margin of error, and an expected proportion of 0.5. The final sample included 189 participants representing all academic years of the program.

The questionnaire was administered online via Google Forms and included items derived from domains recurrently discussed in the student retention and dropout literature.

Participation was voluntary, informed consent was obtained from all respondents, and anonymity and confidentiality were fully guaranteed throughout the data collection and analysis processes.

### 3.2. Measures and Reliability

The measurement instrument was designed to assess five independent variables associated with dropout intention. The questionnaire was developed based on domains recurrently identified in the student retention and dropout literature. Item formulation was discussed among the members of the research team and reviewed by an external expert with experience in scale development and psychometrics in order to assess the conceptual relevance, clarity, and coherence of the items. In addition, a small pilot application was conducted with 15 students to verify item comprehension and the general functioning of the questionnaire before its final administration. Internal consistency was evaluated using Cronbach’s alpha coefficient, which estimates scale reliability based on inter-item correlations. Values of 0.70 or higher are commonly interpreted as acceptable for research purposes.

The dependent variable was measured using a dichotomous item asking students whether they had ever considered leaving the academic program before completion (Larroucau, 2015). Responses were coded as Yes = 1 and No = 0. Although this item provides a narrower operationalization than a multi-item or graded intention scale, it was used in this study as a concise self-reported indicator of withdrawal consideration.

The independent variables were measured using five-point Likert scales (1 = strongly disagree, 5 = strongly agree) and grouped into five variables: personal self-regulation, mental health, institutional perception, socio-economic conditions, and academic motivation. For each variable, item responses were averaged to compute a composite score. In the socio-economic block, all items were negatively worded and referred to financial pressure or economic vulnerability. These items were individually reverse-scored before computing the composite, so that higher values indicate more favorable socio-economic conditions (i.e., lower financial pressure). Because all questionnaires were completed in full, composite scores were computed from complete item responses for all participants.

All multi-item variables demonstrated acceptable to high levels of internal consistency, with Cronbach’s alpha values ranging from 0.712 to 0.845, supporting the reliability of the measures used in subsequent analyses.

Table 1 presents the operational definition, measurement scale, number of items, and reliability coefficient for each variable. Detailed item wording and questionnaire structure for each variable are provided in Appendix A, Table A1.

### 3.3. Data Analysis

All analyses and visualizations were conducted in Python 3.13 using standard libraries for data management, statistical modeling, machine learning, and visualization. Because all respondents completed the questionnaire in full, no missing data were observed at the item level, in the dependent variable, or in the composite scores for the independent variables included in the analyses; therefore, the full analytic sample was retained.

To address the study objective, a multivariable logistic regression model was estimated using maximum likelihood. The dichotomous withdrawal-consideration item was specified as the dependent variable, while the five independent variables (personal self-regulation, mental health, institutional perception, socio-economic conditions, and academic motivation) were included as predictors. Statistical significance was assessed using two-sided tests at α = 0.05. Results are reported as regression coefficients, odds ratios (OR), and 95% confidence intervals. Multicollinearity was assessed using the variance inflation factor (VIF), and additional diagnostic checks were conducted to examine linearity in the logit and potentially influential observations.

For predictive evaluation, the dataset was partitioned once into training and test sets using a stratified 80/20 split with a fixed random_state to ensure reproducibility, and the same partitions were used for both the logistic regression model and the decision tree classifier. Model performance on the held-out test set was assessed using confusion matrices, accuracy, precision, recall, F1-score, and Area Under the Curve (AUC), with predicted probabilities converted into class labels using a 0.50 threshold.

To complement the parametric specification, a shallow decision tree classifier was estimated using the same predictors. The tree was configured with the Gini impurity criterion, a maximum depth of four levels, and a minimum of ten observations per leaf node. These hyperparameters were specified a priori to obtain a parsimonious and interpretable structure and to reduce the risk of overfitting given the sample size. The decision tree was therefore treated primarily as an exploratory and complementary rule-based tool.

To obtain a more robust internal assessment of predictive performance, both models were additionally evaluated using bootstrap validation with out-of-bag testing (1000 iterations). Accuracy, precision, recall, F1-score, and AUC were summarized across bootstrap replications using mean values and percentile-based intervals.

## 4. Results

### 4.1. Descriptive Statistics

In the analytic sample, 111 students (58.7%) reported having considered leaving the program before completion, whereas 78 students (41.3%) reported that they had not. As shown in Table 2, the composite scores for the independent variables are generally located above the midpoint of the scale, which suggests relatively favorable self-reported perceptions across the measured dimensions. Academic motivation exhibits the highest mean, while mental health records the lowest average. Socio-economic conditions also show the greatest variability, pointing to greater heterogeneity in students’ perceived financial circumstances.

### 4.2. Correlation Analysis

**Figure 1:**
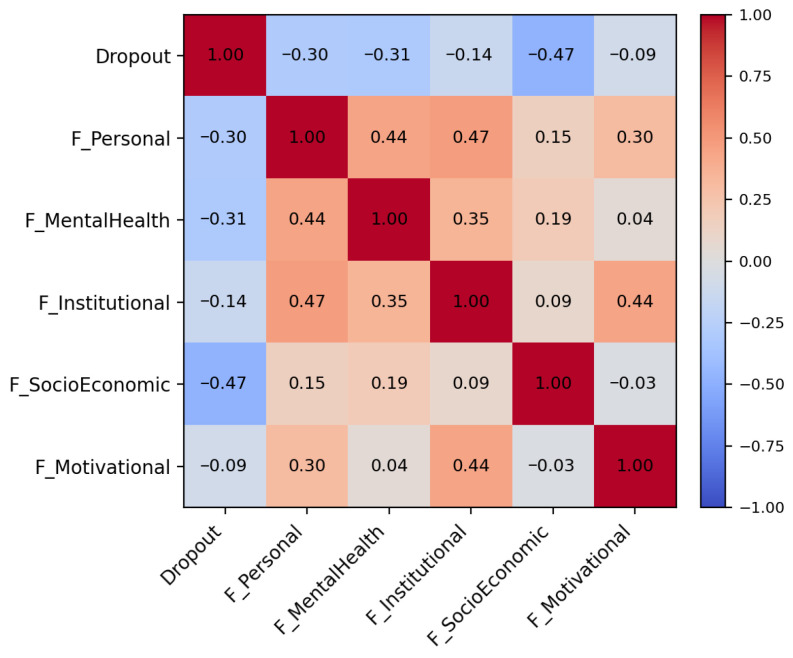
Correlation heatmap between dropout intention and the five explanatory variables.

Figure 1 presents the correlation matrix between dropout intention and the five explanatory variables. Overall, dropout intention exhibits negative associations with three domains: socio-economic conditions, mental health, and personal self-regulation. The strongest correlation is observed for socio-economic conditions, indicating that greater perceived financial stability is associated with a lower likelihood of reporting dropout intention. Moderate negative correlations are also identified for mental health and personal factors. In contrast, institutional perception and academic motivation display comparatively weaker associations with the outcome variable.

### 4.3. Logistic Regression

The multivariable logistic regression model was estimated using the training sub-sample (*n* = 151). The model demonstrates strong global significance (LLR *p*-value = 3.263 × 10^−10^) and satisfactory explanatory capacity (Pseudo R^2^ = 0.269). Estimated coefficients, standard errors, and confidence intervals are presented in Table 3.

Two predictors emerge as statistically significant protective factors. Personal self-regulation (β = −0.937, *p* = 0.005) and socio-economic conditions (β = −0.948, *p* < 0.001) are negatively associated with dropout intention, indicating that higher levels in these domains reduce the likelihood of reporting withdrawal intention. Both variables show similarly strong adjusted protective associations. By contrast, mental health (*p* = 0.193), institutional perception (*p* = 0.324), and academic motivation (*p* = 0.680) do not reach statistical significance after controlling for the remaining predictors.

Odds ratio estimates and multicollinearity diagnostics are summarized in Table 4. Among the predictors with statistical support in the adjusted model, socio-economic conditions (OR = 0.388) and personal self-regulation (OR = 0.392) showed the largest estimated reductions in the odds of dropout intention. Given the similarity of these estimates, the results suggest that both domains are comparatively important protective dimensions in the adjusted model. By contrast, mental health (OR = 0.689), institutional perception (OR = 1.453), and academic motivation (OR = 0.869) do not show statistically supported independent associations with dropout intention. VIF values range from 1.05 to 1.55, indicating no evidence of problematic multicollinearity.

Additional diagnostic checks did not indicate clear violations of linearity in the logit for the continuous independent variables. They also suggested no widespread problems of influential observations. One case showed comparatively higher leverage, Cook’s distance, and standardized residual values, indicating a potentially influential observation; however, no evidence suggested substantial distortion of the coefficient pattern or the overall substantive interpretation. Taken together, these diagnostics suggest that the logistic model provides a reasonably stable basis for interpreting the adjusted associations.

**Figure 2:**
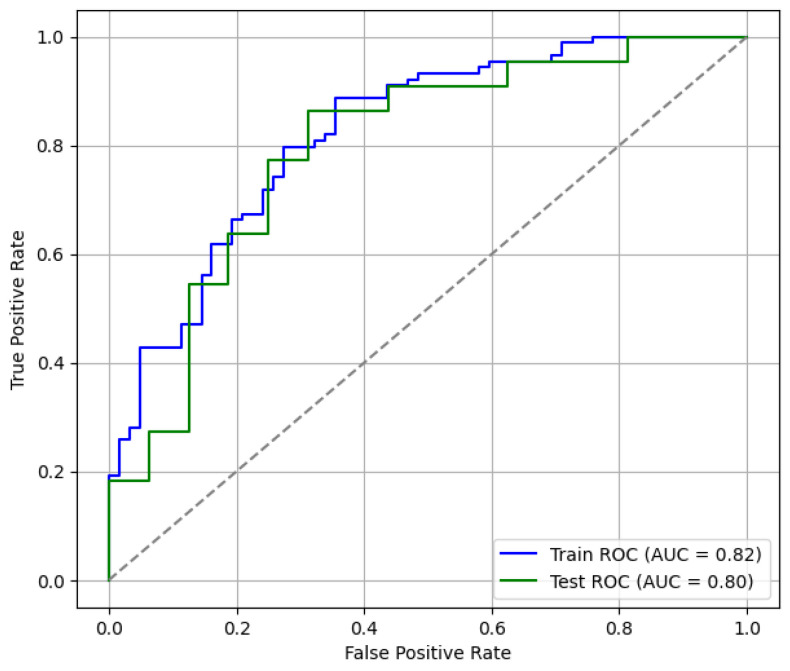
Logistic regression ROC curves (train vs. test).

**Figure 3:**
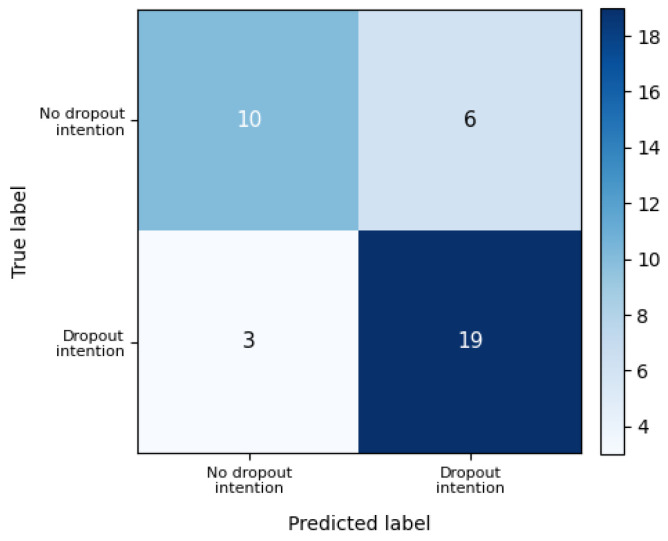
Logistic regression confusion matrix (test set).

Predictive performance was evaluated using the hold-out test set. Figure 2 displays the ROC curves for both training and test samples, illustrating the model’s discriminatory capacity. The confusion matrix derived from the test set classification is presented in Figure 3, providing a detailed view of correct and incorrect classifications.

Classification performance metrics are summarized in Table 5. The single hold-out test set yielded an accuracy of 0.763, precision of 0.760, recall of 0.864, and an F1-score of 0.809. Bootstrap validation, however, produced slightly more conservative mean estimates, suggesting that the logistic regression model retains acceptable and comparatively stable predictive performance across resamples.

### 4.4. Decision Tree

**Figure 4:**
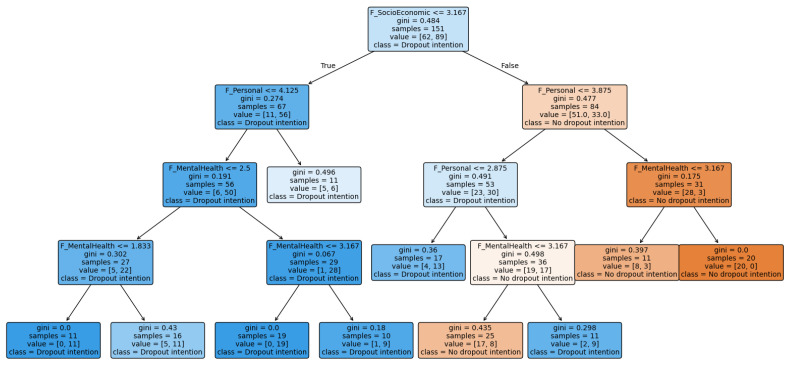
Decision tree trained to predict dropout intention (training set).

To complement the parametric specification, a decision tree classifier was estimated using the same training dataset (*n* = 151). The resulting structure is visualized in Figure 4. The tree reveals a hierarchical segmentation of dropout intention risk, with socio-economic conditions forming the root split, followed by personal self-regulation and mental health in subsequent branches.

**Figure 5:**
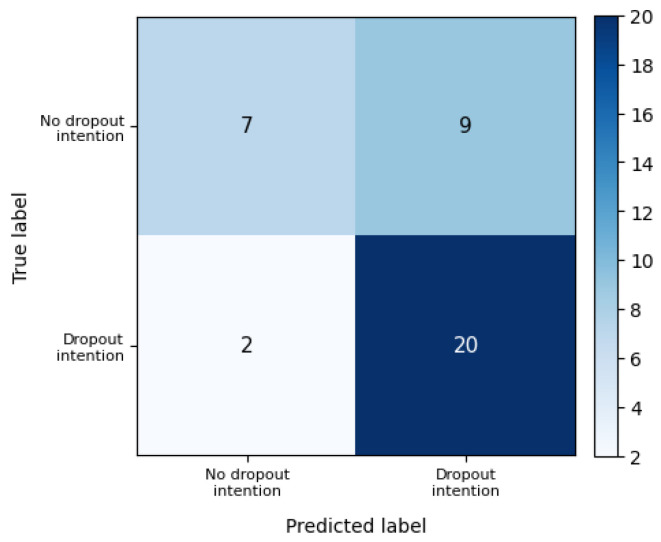
Decision tree confusion matrix (test set).

**Figure 6:**
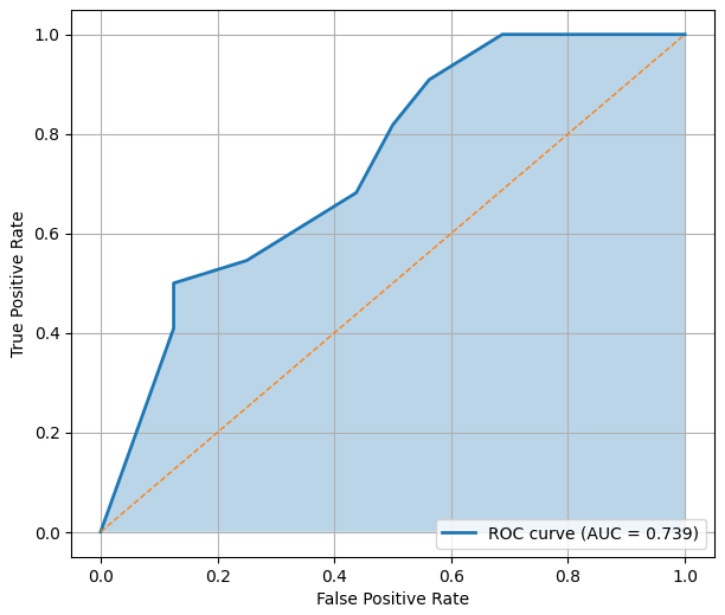
Decision tree ROC curve (test set).

Predictive evaluation on the test set indicates acceptable discriminatory performance. The confusion matrix presented in Figure 5 illustrates the distribution of correct and incorrect classifications produced by the tree model. Figure 6 displays the corresponding ROC curve, providing a visual assessment of model discrimination.

Classification performance metrics are summarized in Table 6. Although the decision tree achieved high recall on the hold-out test set, bootstrap validation indicates greater variability across resamples, supporting its interpretation primarily as an interpretable segmentation tool rather than as a more stable predictive model.

## 5. Discussion

The present study examined dropout intention among undergraduate engineering students through an interpretable modeling approach that considered psychosocial, socio-economic, institutional, and motivational domains simultaneously. Overall, the results indicate that, within the estimated multivariable model, socio-economic conditions and personal self-regulation show the strongest adjusted associations with dropout intention. This pattern is consistent with persistence and attrition frameworks that treat withdrawal as a multidimensional process shaped by the interaction of external constraints, individual adjustment resources, and students’ academic experience rather than by isolated variables alone (J. P. Bean, 1980; Cabrera et al., 1993; Tinto, 1975). In this context, dropout intention appears to be linked more clearly to structural pressures and students’ capacity to regulate academic demands than to institutional or motivational domains considered in isolation.

Among the predictors with statistical support in the adjusted model, socio-economic conditions and personal self-regulation emerged as the two domains most clearly associated with lower dropout intention. This reinforces the view that financial pressure is not merely a background condition, but a central dimension shaping students’ persistence decisions (Aina et al., 2022; Valencia-Arias et al., 2026). Economic strain may reduce the time, concentration, and emotional availability required for sustained academic engagement, especially when students must combine study with employment or face difficulties covering educational expenses. At the same time, self-regulation appears relevant because it helps students organize competing demands, maintain effort, and adapt to the pressures of university life (Bardach et al., 2020; González & Arce, 2021).

Regarding RQ3, the lack of statistical significance for mental health, institutional perception, and academic motivation in the multivariable model should not be interpreted as evidence of irrelevance. A more plausible interpretation is that their contribution may be partly indirect, theoretically more distal, or conceptually adjacent to the domains that show stronger adjusted associations in the present model. In the case of institutional perception and academic motivation, prior work suggests that perceived instructional quality, institutional support, and motivational orientation may influence dropout intention through mediating mechanisms such as belonging, satisfaction, or motivational regulation, rather than always appearing as strong independent predictors in adjusted models (J. Bean & Eaton, 2001; Hadjar et al., 2023; López-Angulo et al., 2024; Wild & Grassinger, 2023). Mental health may likewise remain relevant even when it does not retain statistical significance in the adjusted model, since recent evidence indicates that mental health difficulties continue to play an important role in dropout and dropout intention in higher education, including in engineering-related settings (Del Savio et al., 2022; Zając et al., 2024).

This pattern does not suggest problematic multicollinearity, as VIF remained low. Instead, it indicates that these variables make a more limited unique adjusted contribution within a model that also includes variables more directly related to students’ day-to-day academic functioning and structural conditions. This may be especially relevant in the present context, which is limited to students from a single engineering program at one university. At the same time, the difference between the logistic regression and the decision tree suggests that the role of some domains depends partly on the analytical lens applied. Whereas the logistic model identifies the variables with the strongest independent adjusted contribution, the tree indicates that mental health may still play a secondary role in risk segmentation. Overall, mental health, motivation, and institutional perception remain theoretically relevant, but in this sample, their net explanatory weight appears lower than that of socio-economic conditions and personal self-regulation.

From a methodological perspective, the combined use of multivariable logistic regression and a shallow decision tree is a strength of the study. The logistic model estimates adjusted associations, whereas the decision tree provides an intuitive view of hierarchical predictor patterns. Bootstrap validation further suggests that logistic regression offers more stable predictive performance across resamples, while the tree is more variable, particularly in recall. This supports interpreting the tree mainly as an exploratory and interpretable segmentation tool rather than as a stronger predictive model. More broadly, the study highlights the value of interpretable modeling for comparing the relative explanatory weight of theoretically relevant domains and informing support-oriented decision making in higher education.

Beyond their group-level explanatory value, the findings also speak to research on individual-level prediction and early identification of students at risk of withdrawal. In higher education, predictive analytics and early warning systems increasingly rely on student-level data to support timely interventions, while recent studies stress the importance of combining predictive performance with interpretability (Delogu et al., 2024; Matz et al., 2023; Shafiq et al., 2022; Sohail et al., 2022). From this perspective, socio-economic conditions and personal self-regulation appear especially informative for future risk detection in engineering education. However, early identification should not rely on predictive accuracy alone, since models are more useful when they provide interpretable and actionable signals for support decisions and preventive intervention. Although the present study does not develop a fully operational early warning system, it identifies interpretable domains that may inform future prediction-oriented support efforts. This may be especially relevant because the institutional uptake and sustainability of such practices depend on organizational learning processes and program-level coordination (Pedraja-Rejas et al., 2026).

The transferability of the present findings should be interpreted in light of the specific context in which the study was conducted. The analysis focuses on a single industrial engineering program at a regional Chilean university, and prior research suggests that institutional quality, support structures, and differences across universities may shape students’ persistence conditions in different ways (Larroucau, 2015; Opazo et al., 2021; Sanhueza Gutiérrez et al., 2021). The engineering context may also intensify the relevance of some of the observed relationships, since prior work in engineering education points to demanding academic trajectories, adjustment pressures, and the importance of mental health, academic achievement, and support experiences in students’ withdrawal processes (Casanova et al., 2021; Del Savio et al., 2022). These results should also be understood within the broader Chilean higher education context, characterized by institutional heterogeneity and policy efforts to widen access, even though retention patterns remain uneven across institutions and student groups (Espinoza et al., 2022; OECD, 2017; Opazo et al., 2021). For this reason, the present findings may be more readily transferable to programs and universities with similar student composition, academic demands, and support conditions than to higher education settings in general.

## 6. Conclusions

This study provides interpretable evidence that dropout intention among undergraduate engineering students is most clearly associated with socio-economic conditions and personal self-regulation in the adjusted multivariable model. These findings highlight the relative salience of structural conditions and self-regulatory resources within this specific educational setting. At the same time, the lack of statistical significance for mental health, institutional perception, and academic motivation should not be interpreted as evidence of irrelevance, but rather as suggesting a lower net adjusted contribution in this sample and model.

Several limitations should be acknowledged. The cross-sectional design does not allow causal inference or the examination of changes in dropout intention over time. In addition, the use of self-reported data from a single engineering program at one university limits external generalizability and may introduce reporting bias. Although the sample size was adequate for the analyses conducted, it may still have reduced the ability to detect smaller net effects or more complex patterns of association. Measurement limitations should also be considered. Although the questionnaire was developed based on the student retention and dropout literature, reviewed by an external expert, and piloted with students, no formal exploratory or confirmatory factor analysis was conducted. Accordingly, the structural validity of the measurement model should be interpreted with caution. In addition, dropout intention was measured using a single dichotomous self-report item, which provides a relatively narrow operationalization of a complex construct and limits both measurement precision and construct validity compared with multi-item or graded measures.

Future research should extend this work through longitudinal designs, broader institutional and disciplinary comparisons, and analytical strategies capable of examining interaction effects and subgroup heterogeneity. Such developments would help clarify whether the relative influence of structural, self-regulatory, psychosocial, institutional, and motivational domains remains stable across contexts and over time.

## Figures and Tables

**Table 1:** Variables associated with dropout intention.

Variables	Operational Definition	Number of Items	Cronbach’s α
Dropout Intention	Student’s self-reported consideration of leaving the program before completion	1	–
Personal Self-Regulation	Perceived adaptation to university life, balance between personal and academic responsibilities, and clarity of goals and professional expectations	4	0.712
Mental Health	Perceived ability to manage academic workload and control stress without compromising psychological well-being	3	0.777
Institutional Perception	Evaluation of teaching quality, curriculum design, infrastructure, academic processes, and institutional organization	8	0.845
Socio-Economic Conditions	Perceived economic pressure affecting persistence, including need to work and difficulties financing studies	3	0.743
Academic Motivation	Level of academic effort, engagement, goal orientation, and professional projection	6	0.785

**Table 2:** Descriptive statistics for the independent variables in the analytic sample (*n* = 189).

Variable	Mean	SD	Min	Max
F_Personal	3.417	0.779	1.250	5
F_MentalHealth	2.845	0.907	1	5
F_Institutional	3.251	0.726	1	5
F_SocioEconomic *	3.349	1.168	1	5
F_Motivational	3.687	0.695	1	5

* The socio-economic conditions items were reverse-scored so that higher values indicate more favorable socio-economic conditions (i.e., lower perceived financial pressure and vulnerability).

**Table 3:** Logistic regression results (training set, *n* = 151).

Variable	Coef.	Std. Err.	z	*p*-Value	CI 2.5%	CI 97.5%
Const	7.283	1.635	4.454	<0.001	4.078	10.488
F_Personal	−0.937	0.335	−2.797	0.005	−1.593	−0.280
F_MentalHealth	−0.373	0.287	−1.301	0.193	−0.935	0.189
F_Institutional	0.374	0.379	0.987	0.324	−0.368	1.115
F_SocioEconomic	−0.948	0.197	−4.822	<0.001	−1.333	−0.562
F_Motivational	−0.140	0.340	−0.413	0.680	−0.808	0.527

**Table 4:** Odds ratios and multicollinearity diagnostics (training set, *n* = 151).

Variable	Odds Ratio	VIF
F_Personal	0.392	1.490
F_MentalHealth	0.689	1.350
F_Institutional	1.453	1.550
F_SocioEconomic	0.388	1.049
F_Motivational	0.869	1.304

**Table 5:** Logistic regression test metrics.

Metric	Hold-Out Test Set	Bootstrap Mean	Bootstrap 95% Interval
Accuracy	0.763	0.734	0.642–0.815
Precision	0.760	0.767	0.638–0.886
Recall	0.864	0.793	0.647–0.925
F1-score	0.809	0.776	0.688–0.857
AUC	0.795	0.794	0.701–0.878

**Table 6:** Decision tree test metrics.

Metric	Hold-Out Test Set	Bootstrap Mean	Bootstrap 95% Interval
Accuracy	0.711	0.685	0.569–0.791
Precision	0.690	0.725	0.600–0.844
Recall	0.909	0.758	0.525–0.933
F1-score	0.784	0.735	0.605–0.833
AUC	0.739	0.735	0.624–0.829

## Data Availability

The dataset is not publicly available due to privacy and ethical restrictions; an anonymized version can be made available by the corresponding author upon reasonable request.

## Abbreviations

The following abbreviations are used in this manuscript:

OR	Odds Ratio
VIF	Variance Inflation Factor
ROC	Receiver Operating Characteristic
AUC	Area Under the Curve

## Appendix A

**Table A1:** Questionnaire.

Variable	Questions
Personal Self-Regulation	1. I managed to balance my studies with my personal and family responsibilities. 2. I find it easy to adapt to the academic demands of university. 3. I have clear goals for my academic and professional development. 4. The degree program meets my academic and professional expectations.
Mental Health	5. I feel that I can adequately manage my academic workload without it affecting my mental health. 6. I can organize my study time effectively without feeling stressed. 7. When I have a lot of assessments, I manage to cope with the stress adequately.
Institutional Perception	8. The university infrastructure is adequate. 9. The instructors in my program explain the content clearly and comprehensively. 10. The teaching assistants contribute effectively to my learning and academic development. 11. The assessments fairly reflect the knowledge I acquire. 12. The curriculum for my program is in line with my expectations and professional needs. 13. Communication with lecturers and tutors facilitates my learning and helps me resolve any doubts. 14. Class schedules, assessments and activities are well organized and consistent. 15. The university has clear and fair processes for assessments, enrollment, and academic procedures.
Socio-Economic Conditions	16. I have thought about leaving university for financial reasons. 17. I have considered temporarily leaving university to work and save money. 18. I have to work outside university to cover my expenses.
Academic Motivation	19. I strive to improve my academic performance, even when the results are not as expected. 20. I attend classes and participate actively because I enjoy the learning process. 21. I set challenging academic goals for myself and work constantly to achieve them. 22. Even when I don’t like a subject, I try to perform well out of personal commitment. 23. My classmates and teachers have a positive influence on my motivation to study. 24. I am motivated by the job opportunities my degree offers.
Dropout Intention	25. Have you ever considered leaving the program before completing your studies?
